# Predictive Model for High Coronary Artery Calcium Score in Young Patients with Non-Dialysis Chronic Kidney Disease

**DOI:** 10.3390/jpm11121372

**Published:** 2021-12-15

**Authors:** Tae Ryom Oh, Su Hyun Song, Hong Sang Choi, Sang Heon Suh, Chang Seong Kim, Ji Yong Jung, Kyu Hun Choi, Kook-Hwan Oh, Seong Kwon Ma, Eun Hui Bae, Soo Wan Kim

**Affiliations:** 1 Department of Internal Medicine, Chonnam National University Hospital, Gwangju 61469, Korea; tryeomoh@hanmail.net (T.R.O.); sudang_@naver.com (S.H.S.); hongsang38@hanmail.net (H.S.C.); medssh1984@gmail.com (S.H.S.); laminion@hanmail.net (C.S.K.); drmsk@hanmail.net (S.K.M.); baedak76@gmail.com (E.H.B.); 2 Department of Internal Medicine, Gachon University of Medicine and Science, Incheon 21565, Korea; jyjung@gachon.ac.kr; 3 Department of Internal Medicine, Institute of Kidney Disease Research, College of Medicine, Yonsei University, Seoul 03722, Korea; Khchoi6@yuhs.ac; 4 Department of Internal Medicine, College of Medicine, Seoul National University , Seoul 03080, Korea; ohchris@hanmail.net

**Keywords:** random forest, prediction, coronary artery calcification, machine learning, artificial intelligence, chronic kidney disease

## Abstract

Cardiovascular disease is a major complication of chronic kidney disease. The coronary artery calcium (CAC) score is a surrogate marker for the risk of coronary artery disease. The purpose of this study is to predict outcomes for non-dialysis chronic kidney disease patients under the age of 60 with high CAC scores using machine learning techniques. We developed the predictive models with a chronic kidney disease representative cohort, the Korean Cohort Study for Outcomes in Patients with Chronic Kidney Disease (KNOW-CKD). We divided the cohort into a training dataset (70%) and a validation dataset (30%). The test dataset incorporated an external dataset of patients that were not included in the KNOW-CKD cohort. Support vector machine, random forest, XGboost, logistic regression, and multi-perceptron neural network models were used in the predictive models. We evaluated the model’s performance using the area under the receiver operating characteristic (AUROC) curve. Shapley additive explanation values were applied to select the important features. The random forest model showed the best predictive performance (AUROC 0.87) and there was a statistically significant difference between the traditional logistic regression model and the test dataset. This study will help identify patients at high risk of cardiovascular complications in young chronic kidney disease and establish individualized treatment strategies.

## 1. Introduction

Chronic kidney disease (CKD) is a major health problem, both worldwide and in Korea. When CKD progresses to end stage kidney disease, it causes a heavy socioeconomic burden on both individual patients and communities [1,2]. Among the various complications of CKD, cardiovascular disease (CVD) is at least the second most common cause of death for all stages of CKD patients, and it is the most common cause of death for CKD patients in stages 3–5 [3]. Therefore, in CKD patients, CVD risk assessment and timely intervention may improve the prognosis for CKD patients. Furthermore, the evaluation of CVD risk in younger patients is particularly important. Because younger patients often are more involved in socioeconomic activities than older patients, the development of CVD in young patients has a greater adverse effect on society.

CKD increases the risk of atheromatosis, and it can progress to atherosclerosis [4,5]. The traditional risk factors for CVD in CKD patients include age, hypertension, high fasting glucose, dyslipidemia, and smoking history [6,7,8]. Coronary computed tomography (CT), which is a non-invasive method for evaluating atherosclerosis of the coronary arteries, has been widely used to assess CKD. Coronary CT can calculate a patient’s coronary artery calcium (CAC) score, which is a marker of subclinical coronary artery disease, by measuring the amount of CAC [9,10,11]. However, the use of coronary CT is limited in developing countries due to its high cost, and the effects of radiation exposure prohibit its excessive use.

Recently, many studies have been conducted that apply various machine learning techniques to clinical problems. Prediction models using machine learning techniques have demonstrated better performance than traditional prediction models, such as scoring systems for critical care [12] and traditional statistical models [13]. However, to the best of our knowledge, there has been no study examining the prediction of CAC scores in young non-dialysis CKD patients using machine learning. Therefore, the purpose of this study is to develop a predictive model using machine learning techniques that can screen high-risk patients with coronary artery disease among young chronic kidney disease, and we also compared the performance of machine learning techniques and traditional logistic regression.

## 2. Materials and Methods

### 2.1. Data Source and Study Population

**Figure 1:**
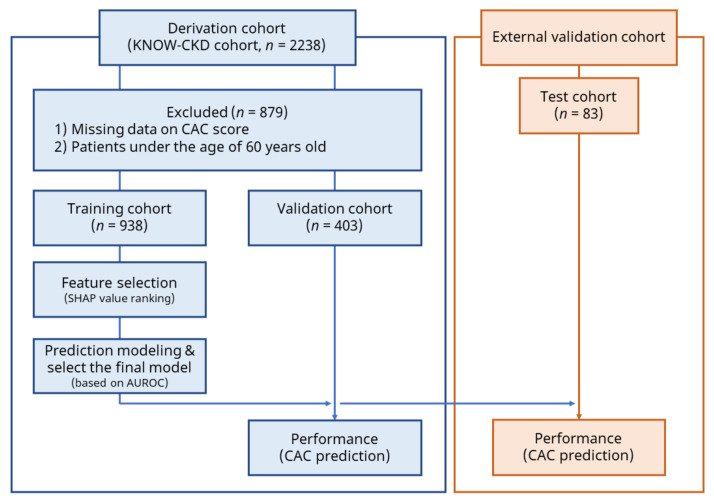
Flow chart representing the workflow of the machine learning analysis. We utilized the derivation cohort to construct the predictive models and external validation cohort to assess the universality of final predictive model. Abbreviations: AUROC, area under the receiver operator curve; CAC, coronary artery calcium; KNOW-CKD, Korean Cohort Study for Outcomes in Patients with Chronic Kidney Disease; SHAP, Shapley additive explanations.

We analyzed data from the Korean Cohort Study for Outcomes in Patients with Chronic Kidney Disease (KNOW-CKD), a nationwide, multicenter prospective cohort study that included non-dialysis patients with stage 1–5 CKD, aged 20–75 years. The detailed methods and design of the study were published previously (NCT01630486 at http://www.clinicaltrials.gov, accessed on 14 December 2021) [14]. The KNOW-CKD cohort included a total of 2238 patients. We excluded 879 patients who had missing CAC scores or were over the age of 60 years old. The final derivation cohort comprised 1341 patients. In addition, we established an external cohort based on patients who were treated at Chonnam National University Hospital for external validation. A total of 83 patients with CKD who were under the age of 60 years old were included in the external validation cohort. The enrollment of patients in this study is summarized in Figure 1.

### 2.2. Measurement and Definition

Various factors are known to be associated with coronary artery calcification; we selected a representative sample of 35 features that were related with coronary artery calcification and used them for the analysis. The details of the selected features are summarized in Table S1. Demographic and baseline clinical data, including age, sex, smoking history, cause of CKD, economic status, educational status, comorbidities, and medication history, were surveyed by well-trained research coordinators. Blood pressure was measured using an electronic sphygmomanometer in the clinic after five minutes of seated rest. Venous blood samples were collected after an overnight fast. Serum creatinine was measured using the traceable isotope-dilution mass spectrometry method. The estimated glomerular filtration rate was calculated using the Chronic Kidney Disease Epidemiology Collaboration (CKD-EPI) equation [15]. First-voided urine was used to measure spot urinary metrics, such as protein and creatinine. Coronary multi-detector CT was performed to calculate the coronary calcium score. The quantitative CAC score was calculated using the method described by Agatston et al. [16]. The primary outcome variable of this study was high CAC score, which was defined as a CAC score ≥100, and the patients were classified based on this criterion.

### 2.3. Statistical Analysis

The data were analyzed using the R language (R Foundation for Statistical Computing, Vienna, Austria, version 4.0.2, http://www.r-project.org, accessed on 14 December 2021) and the Python programming language (Python software foundation, CA, USA, version 3.7, accessed on 14 December 2021). The packages used for the machine learning were: Scikit-learn (version 0.16.1, https://github.com/scikit-learn/scikit-learn, accessed on 14 December 2021) [17], XGboost (The XGBoost Contributors, NY, USA, version 1.4.0, https://github.com/dmlc/xgboost, accessed on 14 December 2021) [18], and Keras (version 2.2.4, https://github.com/keras-team/keras, accessed on 14 December 2021) [19]. We used Scikit-learn packages for the support vector machine (SVM) and Random Forest (RF). XGboost was used for the extreme gradient boosting models and Keras was used for the multilayer perceptron (MLP) neural network models. We also used R for logistic regression with stepwise backward eliminations using Akaike information criterion.

To maintain a constant ratio of primary outcomes in both the training and test datasets, we divided the full dataset into a training dataset (70%) and a validation dataset (30%) using a stratified sampling method. The training dataset was used for developing the predictive models, whereas the validation dataset was used to validate and compare the models. To survey the optimal hyperparameters for machine learning techniques, a 10-fold cross-validation was performed. We used a grid search to investigate the combination of hyperparameters and defined the hyperparameter with the highest the area under the receiver operator curve (AUROC) value as the optimal hyperparameter. We used the AUROC as our main evaluation metric because it features class skew independence, and it is classification-threshold-invariant. The test dataset was utilized only in the performance tests for the final predictive model.

For the neural network model and the SVM, all the variables were normalized with the minimum and maximum values of each variable in the training dataset. The mathematical expressions of the normalization are depicted below:

(1) $$\mathrm{normalization}:z=\frac{x-\min\left(x\right)}{\max\left(x\right)-\min\left(x\right)}$$

We also created dummy features of the discrete variables for appropriate analyses. We calculated the AUROC to quantify the performance of the predictive models and applied the DeLong test to compare the performance of each predictive model. Because missing values cannot be used in machine learning, simple imputation was performed using the MICE package in R [20]. The continuous variables were imputed using the pmm (predictive mean matching) method, the binary variables were imputed by the logreg (logistic regression) method, and the multinomial variables were imputed by the polyreg (polytomous logistic regression) method. Any *p*-values < 0.05 were considered as statistically significant. The Shapley additive explanations (SHAP) value was calculated to determine feature importance. SHAP is based on game theory [21] and local explanations [22]. Lundberg and Lee [23] reported the SHAP value for an explainable model with additive feature attribution methods. Additive feature attribution methods were defined as follows [23]:

(2) $$g\left(z^{\prime }\right)=\;\Phi_{0}+\overset{M}{\underset{i=1}{\sum }}\Phi_{i}{z^{\prime }}_{i}$$

where *z*′ ∈ {0, 1}*^M^*, *M* is the number of input features, and ϕ*_i_* ∈ R.

An important property of the class of additive feature attribution methods is that it has a single unique solution with three desirable properties: local accuracy, omission, and consistency [23]. Based on above method, the authors suggested Tree SHAP, which uses a conditional expectation rather than a marginal expectation [24]:

(3) $$\Phi_{i}=\underset{S\subseteq N\backslash \left\{i\right\}}{\sum }\frac{\left|S\right|!\left(M-\left|S\right|-1\right)!}{M!}\left[f_{x}\left(S\cup \left\{i\right\}\right)-f_{x}\left(S\right)\right]$$

where *N* is the set of all input features

SHAP values can be obtained using the conditional expected value function of the machine learning model, and SHAP utilizes a technique for estimating the Shapley value for the input feature value of each instance [23]. Using SHAP, consistent variable importance can be extracted; Tree SHAP was used in this study.

## 3. Results

### 3.1. Clinical Characteristics of Study Population

The percentages of missing data for all the variables in the derivation cohort were <5%, except for C-reactive protein (7.189%), waist-hip ratio (6.594%), and serum chloride (8.676%), and these missing values were imputed via a simple imputation method using the “MICE” package. The data from 1341 patients were analyzed. The median age and eGFR of the patients in the derivation cohort were 48.0 years and 55.3 mL/min/1.73 m^2^, respectively. The proportion of female patients was 42.4% (568 patients), and the mean waist–hip ratio and fasting blood glucose levels were 0.9 and 107.3 mg/dL, respectively, for the entire derivation cohort. The number of patients with high CAC scores (>100) was 345 (36.8%) in the training dataset and 148 (36.7%) in the validation dataset. Only six features, namely serum albumin, low density lipid, total cholesterol, educational status, serum calcium, and serum phosphate, showed statistical differences between the training and validation cohorts. The detailed characteristics of the study population are summarized in Table S1.

### 3.2. Predictive Models for Coronary Artery Calcium Score

**Figure 2:**
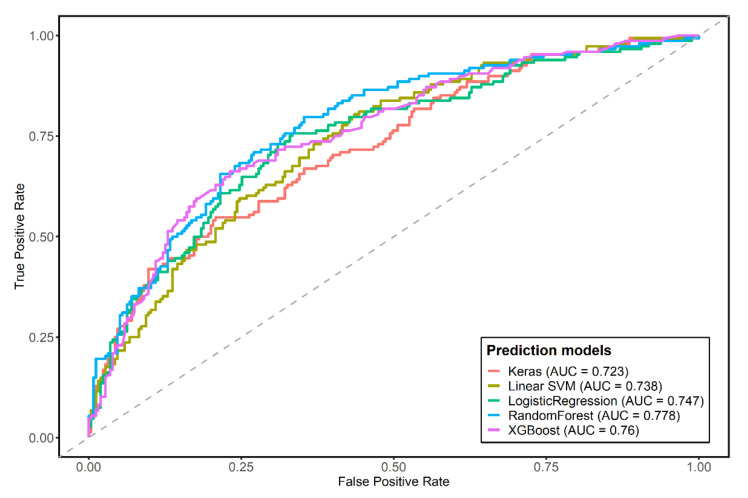
Comparing area under the receiver operator curve of prediction models. The RandomForest model showed the highest area under the receiver operator curve of the coronary artery calcification score prediction. Abbreviations: AUC, area under the receiver operator curve; SVM, support vector machine; XGBoost, extreme gradient boost.

We constructed five predictive models. The summarized results, including the sensitivity, specificity, accuracy, AUROC, and *p*-value for the DeLong test of each predictive model are described in Table 1. The RF and XGboost models showed better accuracy, sensitivity, and specificity than the conventional logistic regression techniques. The SVM and MLP neural network models had lower accuracy, sensitivity, and AUROC than logistic regression. Among the machine learning techniques, the RF model showed the best performance with respect to AUROC and was only statistically significantly different from the performance of logistic regression. We visualize the AUROC in Figure 2. For the logistic regression, Akaike information criterion was applied to select the features using the backward elimination method. The logistic regression data are summarized in Table 2.

### 3.3. Final Predictive Model

**Figure 3:**
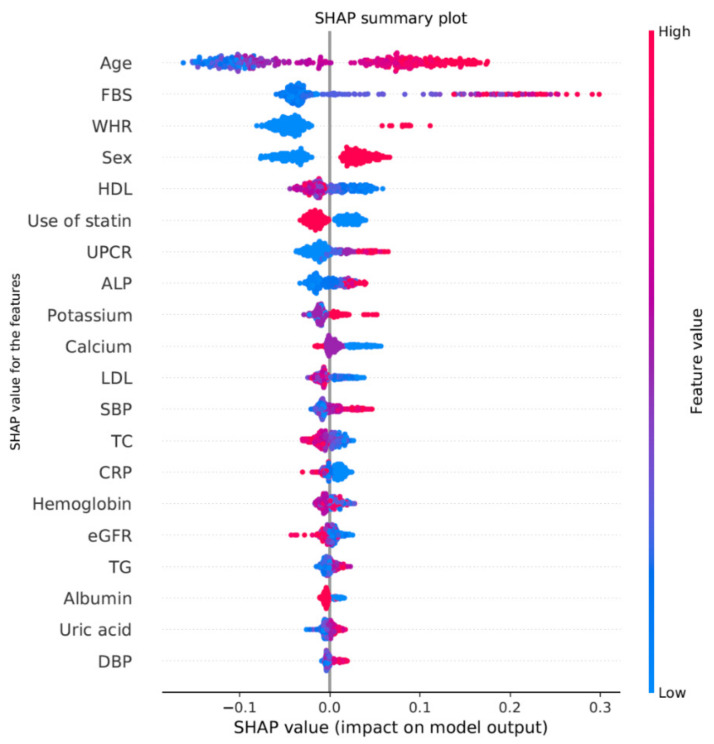
Shapley additive explanations (SHAP) value for RandomForest model. The top 20 features showed the highest SHAP value in the RandomForest model. Abbreviations: SHAP, Shapley additive explanations; FBS, fasting blood glucose; WHR, waist–hip ratio; HDL, high-density lipid; UPCR, urine protein to creatinine ratio; ALP, alkaline phosphatase; LDL, low-density lipid; SBP, systolic blood pressure; TC, total cholesterol; CRP, C-reactive protein; eGFR, estimated glomerular filtration rate; TG, triglyceride; DBP, diastolic blood pressure.

**Figure 4:**
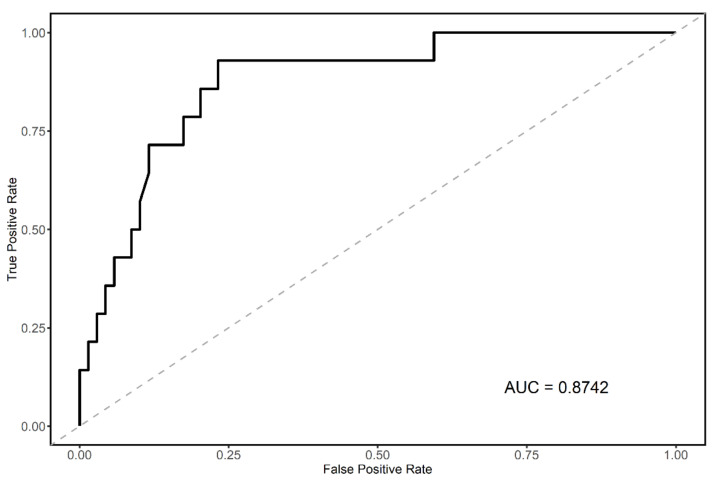
Area under the receiver operator curve of final predictive RandomForest model with test dataset. The area under the receiver operator curve of the final predictive model showed a reliable result. Abbreviation: AUC, area under the receiver operator curve.

The SHAP values of the features in the RF model that showed the best performance were calculated, and the results of the top 20 features are summarized in Figure 3. Age and fasting blood glucose were the highest-ranking features, followed by waist–hip ratio, sex, and high-density lipoprotein. Finally, we selected four features, age, sex, waist-hip ratio, and fasting blood glucose, based on their SHAP values and clinical accessibility. We assessed the performance of the final predictive model with the test dataset, which was independent of the derivation cohort. The AUROC of the final predictive model using the test dataset is visualized in Figure 4, and its AUROC was 0.87.

## 4. Discussion

In this study, we found that traditional logistic regression has limitations in predicting the CAC score of young CKD patients and classifying them into high-risk groups. The RF model showed the best performance with respect to AUROC, requiring only four, easily obtained clinical variables.

Cardiovascular complications are major complications among CKD patients. The selection of high-risk CKD patients for cardiovascular complications and the provision of early interventions are particularly challenging tasks in the medical field. Based on the socioeconomic benefits of early intervention in young patients and the prevalence of high CAC score (>100) increasing with age in Korea [25], we excluded patients over 60 years old. The CAC score is a surrogate marker that can predict the occurrence of cardiovascular events, and it is possible to measure the CAC score using the Agatston method, which measures CAC scores using the weighted sum of lesions with density >130 HU, multiplying the area of calcium by a factor related to maximum plaque attenuation [16]. Recent research has reported that patients with CAC scores >100 have 4.3 times greater risk of experiencing a major cardiovascular event compared to the patients a CAC score of zero [26]. Additionally, the CAC score correlates well with the Framingham risk score, which estimates the 10 year risk of coronary artery disease [27]. Current treatment guidelines recommend that if coronary artery disease is strongly suspected, coronary angiography should be performed first so that both diagnosis and treatment can be performed at the same time. If it is possible to screen patients who are expected to receive a high CAC score, i.e., patients at high risk of coronary artery disease who require coronary angiography. This is very important because unnecessary potentials for double doses of radiation and contrast agents can be avoided.

RF is an ensemble-based tree model that offers the advantage of not easily nor frequently overfitting the data. In addition, rapid performance improvements are not induced by an increase in the training dataset amount, which means that good performances can be generated even with smaller datasets. However, RF features disadvantages in that it has a high computational cost, and it is unable to extract the non-uniform feature importance. To overcome these problems, we constructed the final predictive model by extracting important variables based on the SHAP value and ensured the universality and applicability of the model. Previous reports [28,29] showed the underperformance of predictive models with machine learning techniques in test datasets, which reflects the universality problem of machine learning. However, our final model for predicting high CAC score patients showed a strong predictive performance (0.87 of AUROC) with the external test cohort. This was achieved through careful feature selection.

Currently, machine learning explainability is a major concern, especially in the medical field. Traditional methods (Gini and Split count) for measuring the feature importance of the tree-model feature inconsistent limitations for each model or individual tree. Consistency and accuracy are the important components with which to evaluate the feature importance [19]. Among various methods, SHAP is one of the most reliable technique to assess the feature importance [30]. Our final predictive model comprised only four clinical variables (age, sex, fasting glucose and waist–hip ratio). Known traditional risk factors for CAC include age, high fasting glucose, hypertension, male sex, blood glucose, and waist-hip ratio [7,8,31]. The four variables (age, sex, fasting blood glucose, and waist–hip ratio) that were selected by the SHAP value were consistent with traditional risk factors for CAC, and similar results were observed in logistic regression. Although the SHAP value could not confirm the causality, we concluded that our prediction model is clinically reliable based on these results.

To the best of our knowledge, this study is the first to predict CAC scores based on clinical variables in young non-dialysis CKD patients. Our derivation cohort features many strengths, including its prospective observational design, robust data collection, and large study population. We applied robust statistical methods, including minimized omitted variable bias, with our imputation method and the SHAP value. These strengths ensure that our analyses are reliable. However, our study also features some limitations. First, the database used in this study is larger than in other disease-specific cohorts, but it is smaller than the general population cohort. Second, although the dataset was verified by an external validation cohort, we cannot confirm its universality, which is a limitation of all machine learning methods. Lastly, the study design was retrospective and the problems of hidden bias, confound variables, and omitted variables, which are common to many machine learning techniques, could not be solved completely here.

In this study, our final predictive RF model demonstrated better predictive performance than logistic regression in the assessment of young CKD patients. Our predictive model may help to screen high-risk patients for cardiovascular complications in young chronic kidney disease, without subjecting patients to radiation exposure. Regarding the simplicity and clinical significance of our predictive model, the results of this study may offer great benefits for the efficient use of resources where the use of expensive medical resources, such as CT, is limited. In addition, these results may help in the application of personalized treatment strategies for high-risk patients. In the future, we aim to complete a follow-up study to demonstrate the universality and generalizability of the model.

## Figures and Tables

**Table 1:** Evaluation metrics for prediction models.

	Accuracy	Sensitivity	Specificity	AUROC (95% CI)	*p*-Value for DeLong Test
Logistic regression	0.7022	0.5068	0.8157	0.7467 (0.696–0.797)	reference
XGboost	0.737	0.5405	0.8510	0.7599 (0.711–0.809)	0.3809
RandomForest	0.727	0.5068	0.8549	0.7776 (0.731–0.825)	0.0220
Support vector machine	0.6799	0.17568	0.97255	0.7379 (0.689–0.787)	0.4711
Multilayer perceptron neural network	0.6923	0.4527	0.8314	0.7233 (0.672–0.775)	0.1014

Abbreviations: AUROC, area under the receiver operator curve; CI, confidence interval; XGboost, extreme gradient boost.

**Table 2:** Results of multivariable logistic regression analysis.

Variables	Odds Ratio	Confidence Interval	*p*-Value
Age	1.107	1.081–1.135	<0.001
Male	4.066	2.617–6.405	<0.001
Estimated glomerular filtration rate	1.008	1.001–1.015	0.0174
C-reactive protein	0.941	0.889–0.989	0.0256
Fasting blood glucose	1.01	1.005–1.015	0.0002
High density lipid	0.99	0.978–1.002	0.1219
Total cholesterol	0.995	0.99–1	0.0434
Marital status: Never	1.798	1.043–3.102	0.0345
Marital status: DW	1.515	0.712–3.236	0.2799
Unemployed	1.499	1–2.257	0.0509
Non-use of statin	0.675	0.479–0.952	0.025
Phosphate	1.249	0.945–1.656	0.1207
Waist–hip ratio	39.309	2.587–621.509	0.0086
Hemoglobin	0.921	0.822–1.031	0.1544
Urine protein to creatinine ratio	1.139	1.043–1.248	0.0044
Serum potassium	1.405	0.993–1.992	0.0555

Abbreviation: DW, divorced or widowed.

## Data Availability

The datasets generated during and/or analyzed during the current study are available from the corresponding author (S.W.K.) on reasonable request.

## Supplementary Materials

The following are available online at https://www.mdpi.com/article/10.3390/jpm11121372/s1, Table S1: Clinical characteristics of enrolled patients.

## References

[1] Hunsicker L.G. (2004). The consequences and costs of chronic kidney disease before esrd. J. Am. Soc. Nephrol..

[2] Chadban S.J., Briganti E.M., Kerr P.G., Dunstan D.W., Welborn T.A., Zimmet P.Z., Atkins R.C. (2003). Prevalence of kidney damage in australian adults: The ausdiab kidney study. J. Am. Soc. Nephrol..

[3] Thompson S., James M., Wiebe N., Hemmelgarn B., Manns B., Klarenbach S., Tonelli M. (2015). Cause of death in patients with reduced kidney function. J. Am. Soc. Nephrol..

[4] Reiss A.B., Miyawaki N., Moon J., Kasselman L.J., Voloshyna I., D’Avino R., De Leon J. (2018). Ckd, arterial calcification, atherosclerosis and bone health: Inter-relationships and controversies. Atherosclerosis.

[5] Gracia M., Betriu À., Martínez-Alonso M., Arroyo D., Abajo M., Fernández E., Valdivielso J.M. (2016). Predictors of subclinical atheromatosis progression over 2 years in patients with different stages of ckd. Clin. J. Am. Soc. Nephrol..

[6] Wheeler D.C., Townend J.N., Landray M.J. (2003). Cardiovascular risk factors in predialysis patients: Baseline data from the chronic renal impairment in birmingham (crib) study. Kidney Int..

[7] McCullough P.A., Li S., Jurkovitz C.T., Stevens L., Collins A.J., Chen S.C., Norris K.C., McFarlane S., Johnson B., Shlipak M.G. (2008). Chronic kidney disease, prevalence of premature cardiovascular disease, and relationship to short-term mortality. Am. Heart J..

[8] Nasir K., Santos R.D., Tufail K., Rivera J., Carvalho J.A., Meneghello R., Brady T.D., Blumenthal R.S. (2007). High-normal fasting blood glucose in non-diabetic range is associated with increased coronary artery calcium burden in asymptomatic men. Atherosclerosis.

[9] Greenland P., LaBree L., Azen S.P., Doherty T.M., Detrano R.C. (2004). Coronary artery calcium score combined with framingham score for risk prediction in asymptomatic individuals. JAMA.

[10] O’Malley P.G., Taylor A.J., Jackson J.L., Doherty T.M., Detrano R.C. (2000). Prognostic value of coronary electron-beam computed tomography for coronary heart disease events in asymptomatic populations. Am. J. Cardiol..

[11] Kondos G.T., Hoff J.A., Sevrukov A., Daviglus M.L., Garside D.B., Devries S.S., Chomka E.V., Liu K. (2003). Electron-beam tomography coronary artery calcium and cardiac events: A 37-month follow-up of 5635 initially asymptomatic low- to intermediate-risk adults. Circulation.

[12] Kang M.W., Kim J., Kim D.K., Oh K.H., Joo K.W., Kim Y.S., Han S.S. (2020). Machine learning algorithm to predict mortality in patients undergoing continuous renal replacement therapy. Crit. Care.

[13] Lee H.C., Yoon H.K., Nam K., Cho Y.J., Kim T.K., Kim W.H., Bahk J.H. (2018). Derivation and validation of machine learning approaches to predict acute kidney injury after cardiac surgery. J. Clin. Med..

[14] Oh K.H., Park S.K., Park H.C., Chin H.J., Chae D.W., Choi K.H., Han S.H., Yoo T.H., Lee K., Kim Y.S. (2014). Know-ckd (korean cohort study for outcome in patients with chronic kidney disease): Design and methods. BMC Nephrol..

[15] Miller W.G., Myers G.L., Ashwood E.R., Killeen A.A., Wang E., Thienpont L.M., Siekmann L. (2005). Creatinine measurement: State of the art in accuracy and interlaboratory harmonization. Arch. Pathol. Lab. Med..

[16] Agatston A.S., Janowitz W.R., Hildner F.J., Zusmer N.R., Viamonte M., Detrano R. (1990). Quantification of coronary artery calcium using ultrafast computed tomography. J. Am. Coll. Cardiol..

[17] Pedregosa F., Varoquaux G., Gramfort A., Michel V., Thirion B., Grisel O., Blondel M., Prettenhofer P., Weiss R., Dubourg V. (2012). Scikit-learn: Machine learning in python. J. Mach. Learn. Res..

[18] Chen T., Guestrin C. Xgboost: A scalable tree boosting system. Proceedings of the 22nd ACM SIGKDD International Conference on Knowledge Discovery and Data Mining.

[19] Gulli A., Pal S. (2017). Deep Learning with Keras.

[20] Buuren S., Groothuis-Oudshoorn C. (2011). Mice: Multivariate imputation by chained equations in R. J. Stat. Softw..

[21] Štrumbelj E., Kononenko I. (2013). Explaining prediction models and individual predictions with feature contributions. Knowl. Inf. Syst..

[22] Ribeiro M., Singh S., Guestrin C. “Why should i trust you?”: Explaining the predictions of any classifier. Proceedings of the 22nd ACM SIGKDD International Conference on Knowledge Discovery and Data Mining.

[23] Lundberg S., Lee S.-I. A unified approach to interpreting model predictions. Proceedings of the 31st International Conference on Neural Information Processing Systems.

[24] Lundberg S., Erion G., Lee S.-I. (2018). Consistent individualized feature attribution for tree ensembles. arXiv.

[25] Park H.E., Kim M.K., Choi S.Y., Lee W., Shin C.S., Cho S.H., Oh B.H. (2012). The prevalence and distribution of coronary artery calcium in asymptomatic korean population. Int. J. Cardiovasc. Imaging.

[26] Greenland P., Bonow R.O., Brundage B.H., Budoff M.J., Eisenberg M.J., Grundy S.M., Lauer M.S., Post W.S., Raggi P., Redberg R.F. (2007). Accf/aha 2007 clinical expert consensus document on coronary artery calcium scoring by computed tomography in global cardiovascular risk assessment and in evaluation of patients with chest pain: A report of the american college of cardiology foundation clinical expert consensus task force (accf/aha writing committee to update the 2000 expert consensus document on electron beam computed tomography) developed in collaboration with the society of atherosclerosis imaging and prevention and the society of cardiovascular computed tomography. J. Am. Coll. Cardiol..

[27] Lichtenstein G., Perlman A., Shpitzen S., Durst R., Shaham D., Leitersdorf E., Szalat A. (2018). Correlation between coronary artery calcification by non-cardiac ct and framingham score in young patients. PLoS ONE.

[28] Goldstein B.A., Navar A.M., Carter R.E. (2017). Moving beyond regression techniques in cardiovascular risk prediction: Applying machine learning to address analytic challenges. Eur. Heart J..

[29] Deo R.C. (2015). Machine learning in medicine. Circulation.

[30] Lundberg S., Erion G., Chen H., DeGrave A., Prutkin J., Nair B., Katz R., Himmelfarb J., Bansal N., Lee S.-I. (2020). From local explanations to global understanding with explainable ai for trees. Nat. Mach. Intell..

[31] Oh H.G., Nallamshetty S., Rhee E.J. (2016). Increased risk of progression of coronary artery calcification in male subjects with high baseline waist-to-height ratio: The kangbuk samsung health study. Diabetes Metab. J..

