# Supplementary material for: Predictive Model for High Coronary Artery Calcium Score in Young Patients with Non-Dialysis Chronic Kidney Disease
Source: J Pers Med. 2021 Dec 15;11(12):1372. doi: 10.3390/jpm11121372 (PMC8703324; doi:10.3390/jpm11121372)
Supplement: Supplementary file 1 [file jpm-11-01372-s001.zip › jpm-1507303-supplementary.pdf]

Journal of
Personalized
Medicine

MDPI

Supplementary Materials

Table S1. Clinical characteristics of enrolled patients.

Features	Training	Validation	*p*-value
Age	46.5 ± 9.0	46.3 ± 9.6	0.732
Sex			0.885
Female	399 (42.5%)	169 (41.9%)	
Male	539 (57.5%)	234 (58.1%)	
Estimated glomerular filtration rate	55.6 (32.7;88.2)	54.5 (32.3;85.2)	0.947
Serum albumin	4.2 ± 0.4	4.2 ± 0.4	0.011
Uric acid	6.9 ± 2.0	7.0 ± 1.9	0.657
C-reactive protein	1.7 ± 3.9	2.0 ± 5.4	0.301
Fasting blood glucose	106.7 ± 38.2	108.6 ± 43.7	0.457
Smoking history			0.557
Current	528 (56.3%)	218 (54.1%)	
Never	169 (18.0%)	70 (17.4%)	
Ex	241 (25.7%)	115 (28.5%)	
High density lipid	48.0 (40.0;59.0)	49.0 (39.0;59.5)	0.837
Low density lipid	94.0 (75.0;117.0)	101.0 (80.0;122.0)	0.012
Triglyceride	127.0 (89.0;187.0)	133.0 (92.0;195.0)	0.209
Total cholesterol	172.0 (150.0;199.0)	178.0 (155.0;203.0)	0.032
Educational status			0.026
Below elementary school	46 (4.9%)	32 (7.9%)	
Middle school	88 (9.4%)	23 (5.7%)	
High school	355 (37.8%)	149 (37.0%)	
Above university	449 (47.9%)	199 (49.4%)	
Economic status			0.843
Low	251 (26.8%)	114 (28.3%)	
Middle	545 (58.1%)	230 (57.1%)	
Hight	142 (15.1%)	59 (14.6%)	
Marital status			0.353
Married	747 (79.6%)	334 (82.9%)	
Unmarried	149 (15.9%)	52 (12.9%)	
Divorced or widowed	42 (4.5%)	17 (4.2%)	
Work status			0.847
Employed	656 (69.9%)	279 (69.2%)	
Unemployed	282 (30.1%)	124 (30.8%)	
Use of statin			0.415
Yes	434 (46.3%)	176 (43.7%)	
No	504 (53.7%)	227 (56.3%)	
Use of ezemitide			0.617
Yes	62 (6.6%)	23 (5.7%)	
No	876 (93.4%)	380 (94.3%)	
White blood cell	6559.8 ± 2006.6	6557.3 ± 1920.5	0.983
Calcium	9.1 ± 0.5	9.2 ± 0.5	0.007
Phosphate	3.6 ± 0.7	3.7 ± 0.7	0.042
Serum ferritin	98.0 (51.2;177.5)	97.2 (47.6;169.5)	0.655
Use of ACE inhibitor			0.249
Yes	128 (13.6%)	45 (11.2%)	
No	810 (86.4%)	358 (88.8%)	
Use of ARB			0.971
Yes	747 (79.6%)	322 (79.9%)	
No	191 (20.4%)	81 (20.1%)	

Mean ankle to brachial index	1.1 ± 0.1	1.1 ± 0.1	0.878
Body mass index	24.4 ± 3.6	24.3 ± 3.5	0.667
Systolic blood pressure	126.5 ± 16.0	126.6 ± 15.4	0.953
Diastolic blood pressure	78.4 ± 10.7	78.0 ± 10.9	0.606
Waist hip ratio	0.9 ± 0.1	0.9 ± 0.1	0.7
Hemoglobin	13.0 ± 2.0	13.1 ± 2.0	0.359
Urine protein to creatinine ratio	1.3 ± 2.2	1.1 ± 1.7	0.277
Alkaline phosphatase	67.0 (52.0;89.0)	65.0 (51.0;90.0)	0.666
Serum sodium	140.8 ± 2.4	140.7 ± 2.5	0.504
Serum chloride	105.4 ± 3.6	105.2 ± 3.5	0.303
Serum potassium	4.6 ± 0.5	4.6 ± 0.6	0.899
High coronary artery calcium score			1
No	593 (63.2%)	255 (63.3%)	
Yes	345 (36.8%)	148 (36.7%)	
